# Improved Compliance With Postoperative Neurosurgical SSI Preventative Measures

**DOI:** 10.1097/pq9.0000000000000604

**Published:** 2022-10-03

**Authors:** Lisa Regalado, Yvette Ziesemer-Girouard, Jennifer Rizzi, Brittney Frazier, Sarah L. Hubert, Manish Shah

**Affiliations:** From the * Children’s Memorial Hermann Hospital, Department of Children's Quality, 6411 Fannin St., Houston, TX 77030; † Department of Neurosurgery, University of Texas Health Science Center of Houston 7000 Fannin St., Houston, TX 77030.

## Background:

Pediatric neurosurgical patients at Children’s Memorial Hermann Hospital (CMHH) were experiencing a high rate of surgical site infections (SSIs) at the end of 2020. At this time, the neurosurgical SSI National Healthcare Safety Network Standard Infection Ratio had an overall rate of 1.30 (N = 11), and it was noted that previous work to prevent SSIs in this population had centered specifically on care of the patients preoperatively and intraoperatively versus postoperatively.

## Objectives:

Given that initial efforts to prevent neurosurgical SSIs had focused on preoperative and intraoperative care the objective became to identify postoperative interventions. The interventions would help to create and maintain consistent postoperative care for pediatric neurosurgical patients. The overall goal was to decrease the pediatric neurosurgical SSI Standard Infection Ratio to below 1 by the end of 2021.

## Methods:

A multidisciplinary taskforce made up of peri anesthesia and perioperative nursing staff, infection control, hospital quality, and pediatric neurosurgical providers was developed. The taskforce worked together using the “Voice of the Customer” and a fishbone diagram to identify postoperative nursing interventions, specifically around patient hygiene and cleansing (bathing, shampooing, and surgical site care), to help prevent postoperative neurosurgical SSIs. After educating the bedside staff on the taskforce recommended interventions, there was not a noticeable decrease in the amount of postoperative neurosurgical SSIs. An audit revealed low nursing compliance with the recommended interventions. The taskforce realized an opportunity with this lack of compliance and developed a postoperative pediatric neurosurgical order set/bundle using the identified recommended postoperative interventions. The finalized order set went live in August 2021, after the completion of nursing and provider education. Monthly compliance regarding physician order entry and nursing documentation of ordered interventions started immediately after go-live.

## Results:

**Fig. 1.**
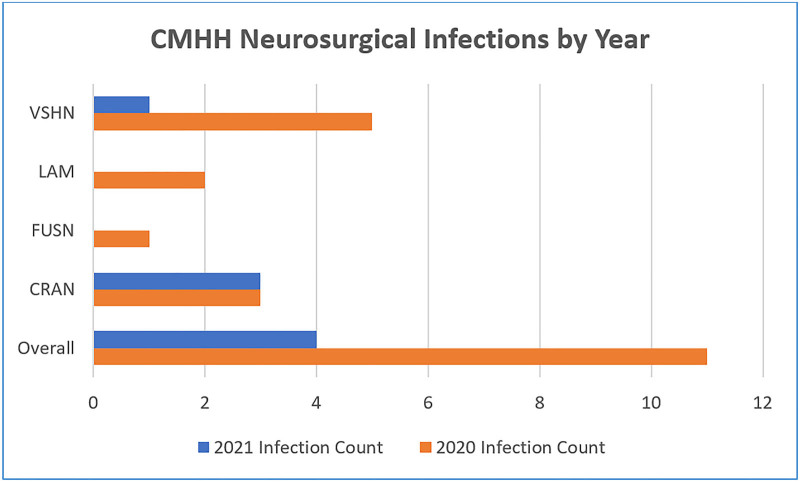
neurosurgical infections by year.

**Fig. 2.**
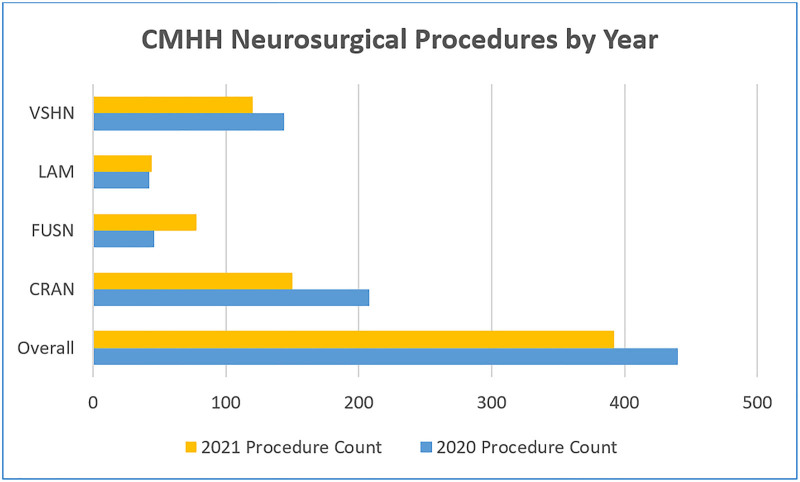
neurosurgical procedures by year.

With the creation and implementation of the postoperative order set, postoperative provider order entry increased from 21% to 63%. There was an overall pediatric neurosurgery SSI rate drop from 1.30 in 2020 to 0.51 in 2021, and the number of cases of infection decreased significantly (Fig. 1) while the number of cases performed stayed relatively the same (Fig. 2).

## Conclusions/Implications:

The work this taskforce did emphasizes how important a postoperative bundle, staff education, and bundle ordering compliance is to helping decrease pediatric neurosurgery SSI rates. Having an established order set will lead to increased compliance with the ordering and implementation of postoperative nursing interventions. In turn, postoperative neurosurgical patients will experience more consistent care across all hospital based units.

## DISCLOSURE

The authors have no financial interest to declare in relation to the content of this article.

